# M. tuberculosis AlkX Encoded by *rv3249c* Regulates a Conserved Alkane Hydroxylase System That Is Important for Replication in Macrophages and Biofilm Formation

**DOI:** 10.1128/spectrum.01969-22

**Published:** 2022-08-08

**Authors:** Haley Stokas, Heather L. Rhodes, Marit B. Simmons, Richard Zhang, Catherine C. Wright, Georgiana E. Purdy

**Affiliations:** a Oregon Health & Science University, Department of Molecular Microbiology & Immunology, Portland, Oregon, USA; Johns Hopkins University School of Medicine

**Keywords:** *Mycobacterium tuberculosis*, transcription factor, alkane hydroxylase, rubredoxin, biofilm

## Abstract

Mycobacterium tuberculosis is a highly specialized human pathogen. The success of M. tuberculosis is due to its ability to replicate within host macrophages, resist host immune responses, and ultimately enter a persistent state during a latent tuberculosis infection. Understanding how M. tuberculosis adapts to and replicates in the intracellular environment of the host is crucial for the development of novel, targeted therapeutics. We report the characterization of an M. tuberculosis mutant lacking Rv3249c, a TetR transcriptional regulator. We show that Rv3249c directly represses the adjacent *alkB-rubA-rubB* operon encoding an alkane hydroxylase/rubredoxin system. For consistency with related systems, we have named the *rv3249c* gene *alkX*. The *alkX* mutant survived better than wild-type M. tuberculosis inside macrophages. This could be phenocopied by overexpression of the *alkB-rubA-rubB* locus. We hypothesized that the improved intracellular survival phenotype is a result of increased fitness of the mutant; however, we found that the *alkX* mutant had a defect when grown on some host-associated carbon sources *in vitro*. We also found that the *alkX* mutant had a defect in biofilm formation, also linked to the overexpression of the *alkB-rubAB* genes. Combined, these results define the primary role of AlkX as a transcriptional repressor of the *alkB-rubAB* operon and suggest the operon contributes to intracellular survival of the pathogen.

**IMPORTANCE** Mycobacterium tuberculosis, the causative agent of tuberculosis (TB), is the leading cause of death worldwide due to a single infectious agent. It is important to understand how M. tuberculosis adapts to and replicates in the intracellular environment of the host. In this study, we characterized the TetR transcriptional regulator Rv3249c and show that it regulates a highly conserved alkane hydroxylase/rubredoxin system. Our data demonstrate that the AlkBRubAB system contributes to the success of the bacterium in host macrophages.

## INTRODUCTION

Mycobacterium tuberculosis, the causative agent of tuberculosis (TB), is the leading cause of death worldwide due to a single infectious agent. The success of M. tuberculosis as a human pathogen can be attributed to its ability to replicate in macrophages, evade host immune responses, and establish a latent infection. Understanding the mechanisms and regulatory processes that underly the ability of M. tuberculosis to survive in the host and establish dormancy is key to the development of novel therapeutics that target latent TB infections.

M. tuberculosis metabolism during infection has been intensely researched. Genetic studies characterizing M. tuberculosis mutants revealed that M. tuberculosis utilizes both host fatty acids and cholesterol as carbon sources when growing intracellularly. Coupling of fatty acid β-oxidation with the glyoxylate cycle is required for successful utilization of fatty acids, as demonstrated by the essentiality of isocitrate lyase 1 and 2 (1). Similarly, the Mce4 cholesterol import system is required for M. tuberculosis virulence (2). Recent work showed that the Mce1 transporter imports fatty acids and that there is interplay between the utilization of these two carbon sources (3). Earlier metabolomic studies also highlight the ability of M. tuberculosis to utilize multiple carbon sources at once (4).

We report the characterization of an M. tuberculosis mutant lacking Rv3249c, a member of the TetR family of transcriptional regulators. We show that Rv3249c represses the operon encoding the alkane hydroxylase AlkB and the rubredoxins RubA and RubB. AlkB is a predicted alkane hydroxylase belonging to a family of widely distributed integral membrane non-heme diiron monooxygenases that permit the utilization of medium- and long-chain (C5-C16) alkanes as a carbon source. AlkB proteins also have homology with membrane-bound fatty acid desaturases (5). M. tuberculosis AlkB has 40% and 44% identity to the characterized alkane hydroxylases from Pseudomonas putida and Pseudomonas aeruginosa, respectively. Using a heterologous expression system, Smits et al. showed that M. tuberculosis AlkB permitted growth of P. putida and P. fluorescens AlkB mutants on C10-12 and C12-16 alkane vapors, demonstrating conserved alkane hydroxylase function (6). However, M. tuberculosis was not able to utilize alkanes *in vitro*, revealing potential differences between the species (7). In addition, AlkB proteins typically require one rubredoxin and a rubredoxin reductase. Rubredoxins are iron-sulfur cluster-containing redox-active proteins that shuttle electrons from alkane hydroxylases. M. tuberculosis RubA and RubB were identified as AlkG1- and AlkG2-type rubredoxins, respectively, based on their ability to complement growth on *n*-octane vapor as a carbon source in a P. putida GPo1 rubredoxin *Rd2* mutant (8). Recent *in vitro* biochemical studies showed that RubB can function as a redox partner for several cytochrome P450 proteins, suggesting a role in cholesterol or fatty acid metabolism (9). The M. tuberculosis gene encoding a rubredoxin reductase has not been identified, and there are no likely candidates in the immediate vicinity of *alkB-rubAB*.

The gene encoding the Rv3249c transcriptional regulator is immediately downstream of the *alkB-rubAB* genes in M. tuberculosis. Since this organization is similar to that in other organisms where the TetR regulator homologue is known as AlkX (10), we named *rv3249c alkX*. We report here that M. tuberculosis AlkX represses *alkB-rubAB* expression. We show that the *alkX* mutant survives better than wild-type M. tuberculosis in macrophages, which can be phenocopied by the overexpression of AlkBRubAB. We show that the *alkX* mutant has impaired biofilm formation, also due to overexpression of AlkBRubAB. These studies define the primary role of AlkX in regulating the AlkBRubAB system.

## RESULTS

### *rv3249c* encodes the transcription factor AlkX, which controls the expression of the *alkB-rubAB* genes.

**FIG 1:**
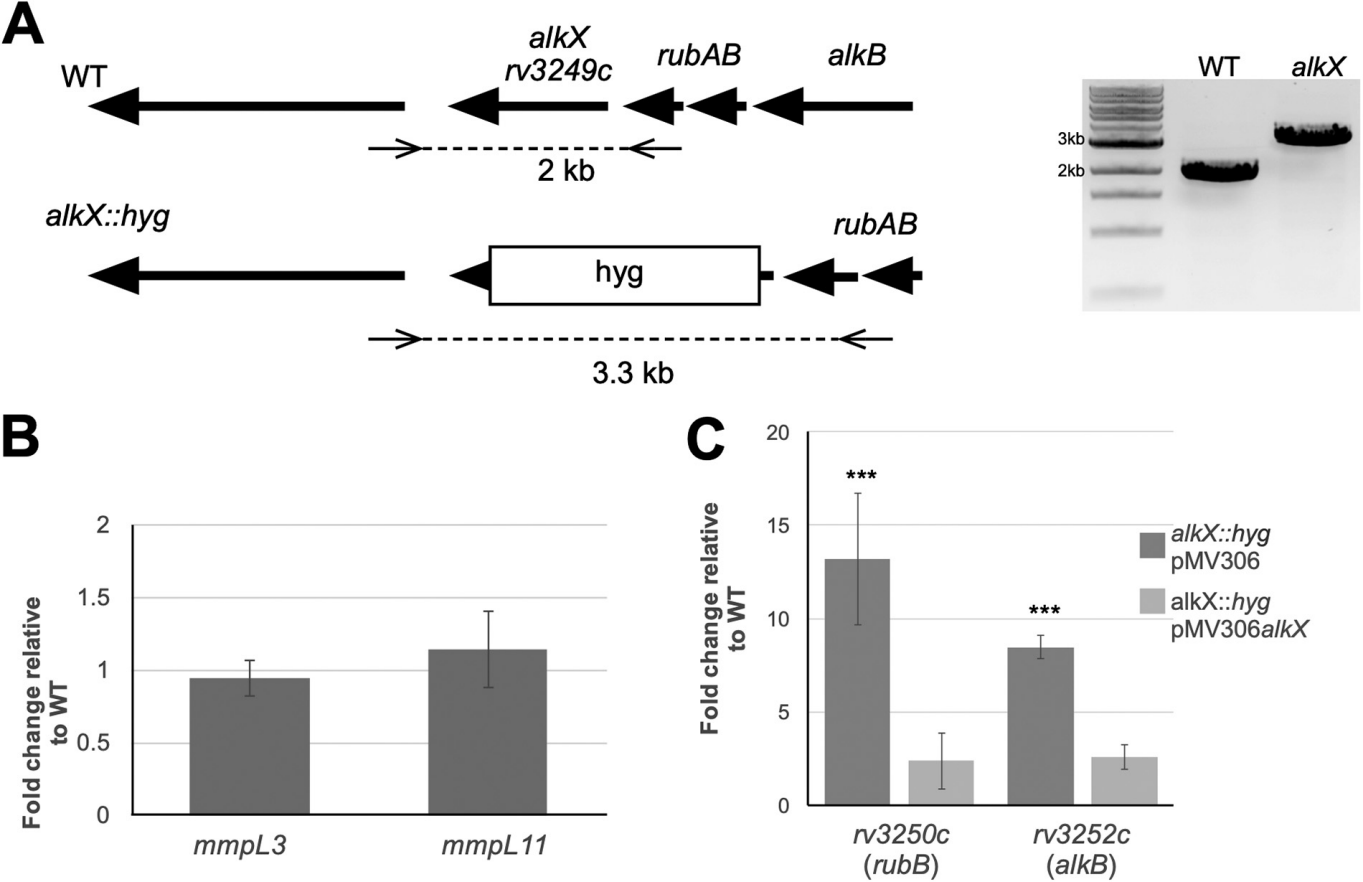
The *alkX* mutant displays increased expression of adjacent *rubA* and *alkB* genes. (A) The *alkX* mutant was constructed by replacing the coding sequence with a hygromycin resistance cassette by allelic exchange in the H37Rv strain background. The mutant *alkX* locus was confirmed via PCR amplification and sequencing. (B) qRT-PCR analysis showed no significant difference in the transcription of *mmpL3* and *mmpL11* between the mutant and wild-type strains. (C) qRT-PCR analysis showed that *rv3250c* (*rubB*) and *rv3252c* (*alkB*) were highly upregulated in the *alkX* mutant relative to wild-type M. tuberculosis. The difference between *rubB* and *alkB* expression in the mutant compared to wild-type M. tuberculosis was significant, Student’s *t* test: ***, *P* < 0.005. In panels B and C, the averages and standard deviation of three biological replicates are shown.

AlkX was identified as a potential regulator of the lipid transporter MmpL proteins in published chromatin immunoprecipitation sequencing (ChIP-seq) studies (11, 12). Using electrophoretic mobility shift assay (EMSAs), we previously showed that AlkX bound to inter- and intragenic regions of the *mmpL3* and *mmpL11* genes (13). To further characterize AlkX, we generated a hygromycin-marked deletion mutant in the M. tuberculosis H37Rv background. The mutation of *alkX* was verified by PCR amplification of the genomic locus followed by sequence analysis (Fig. 1A).

To examine the function of AlkX as a transcriptional regulator of the *mmpL3*/*mmpL11* locus, we quantified expression of *mmpL3* and *mmpL11* in wild-type and *alkX* mutant strains using reverse transcription-quantitative PCR (qRT-PCR). There was no difference in expression of either gene between the wild-type and mutant strains (Fig. 1B). This result likely reflects complex regulation of these genes since we showed that a number of transcription factors bind the promoter or intragenic regions of the *mmpL3*/*mmpL11* locus, including Rv1816, Rv0302, Rv1049, and Rv0687 (13, 14).

To identify the AlkX regulon, we performed RNA-seq of wild-type and *alkX* mutant M. tuberculosis. Using this analysis, we found 41 genes upregulated in the *alkX* mutant compared to wild-type M. tuberculosis (Table 1). Of the upregulated genes, seven encode other transcription factors. ChIP-Seq data indicate that one of these, the transcriptional regulator Rv1990c, is likely directly regulated by AlkX (15). There were 4 genes downregulated in the *alkX* mutant compared to wild-type M. tuberculosis (*rv3738c* and *rv3740c* to *3742c*). The most highly upregulated gene was *whiB7*; however, we did not observe significant differences between wild-type and *alkX* mutant M. tuberculosis when we tried to confirm these results using qRT-PCR, and there is no ChIP-Seq evidence of direct binding to this genomic locus. Interestingly, the genes directly upstream of *rv3249c* comprising *alkB*, *rubA*, and *rubB* were upregulated ~7 to 9-fold. Reexamination of the AlkX ChIP-Seq data revealed a significant peak at chromosomal locus 3632057, which corresponds to the DNA region upstream of *alkB*. Combined, these data suggested that AlkX directly regulates the upstream adjacent genes. We performed qRT-PCR to quantify expression differences in *rubB* and *alkB* between the wild-type and *alkX* mutant strains. Both genes were significantly upregulated in the *alkX* mutant compared to wild-type M. tuberculosis (Fig. 1C). Wild-type expression levels of *rubB* and *alkB* were restored by complementation of the *alkX* mutant via the integrative plasmid pMV306*alkX*. These data suggest that AlkX is a repressor of the adjacent genetic locus consisting of *rv3250* to *3252c*.

**TABLE 1:** Upregulated genes in *alkX* mutant versus wild-type M. tuberculosis

Gene name	Log2 fold change	Protein	Adjusted *P* value
*whiB7*	6.56396615	Transcriptional regulator WhiB7	1.23E-27
*Rv3196A*	3.762293978	Hypothetical protein	1.77E-30
*erm*(*37*)	3.713324331	23S rRNA [adenine(2058)-N(6)]-methyltransferase	7.06E-33
*rubB*	3.253436291	Rubredoxin RubB	3.03E-40
*Rv1258c*	2.939729139	Multidrug-efflux transporter	7.31E-35
*alkB*	2.892022348	Transmembrane alkane 1-monooxygenase AlkB	1.17E-11
*eis*	2.883561582	Enhanced intracellular survival protein	2.23E-16
*rubA*	2.846876423	Rubredoxin RubA	9.06E-21
*mpt70*	2.754519361	Major secreted immunogenic protein Mpt70	7.37E-28
*Rv2876*	2.669310178	Transmembrane protein	5.88E-28
*Rv0263c*	2.668746216	Hypothetical protein	5.26E-35
*Rv0264c*	2.519716243	Hypothetical protein	1.48E-38
*esxP*	2.432838873	ESAT-6 like protein EsxP	4.93E-06
*Rv1265*	2.412824561	Hypothetical protein	3.93E-06
*Rv1460*	2.308803616	Transcriptional regulator	9.18E-10
*ppsC*	2.215838437	Phthiocerol synthesis polyketide synthase type I	5.32E-09
*Rv2034*	2.187145617	ArsR family HTH-type transcriptional repressor	0.001
*ppsB*	2.174928755	Phthiocerol synthesis polyketide synthase type I	1.03E-06
*Rv0691A*	2.062391597	Mycofactocin precursor	0.006
*Rv1815*	2.019448731	Hypothetical protein	0.0001
*Rv0887c*	1.987503287	Hypothetical protein	3.85E-09
*hflX*	1.953454721	GTP-binding protein HflX	3.09E-17
*Rv2253*	1.939661218	Hypothetical protein	0.011
*Rv2348c*	1.907360528	Hypothetical protein	5.54E-08
*mpt83*	1.90467678	Cell surface lipoprotein	3.16E-09
*Rv2415c*	1.893901822	Hypothetical protein	1.90E-44
*Rv2254c*	1.867175632	Integral membrane protein	7.40E-10
*rpfC*	1.833812216	Resuscitation-promoting factor RpfC	0.058
*Rv0576*	1.772721362	Transcriptional regulator	0.005
*cut2*	1.697418879	Cutinase	8.88E-08
*Rv2256c*	1.690773527	Hypothetical protein	6.72E-08
*rslA*	1.659062153	Anti-sigma-L factor RslA	4.51E-11
*sigL*	1.629996056	ECF RNA polymerase sigma factor SigL	0.0001
*PPE15*	1.616075072	PPE family protein PPE15	0.00001
*Rv0449c*	1.580359555	Hypothetical protein	2.04E-07
*Rv2250c*	1.579601193	HTH-type transcriptional regulator	3.16E-09
*proB*	1.55670483	Glutamate 5-kinase protein	1.49E-09
*dipZ*	1.541461013	Integral membrane C-type cytochrome biogenesis protein	0.008
*Rv2828c*	1.53964006	Hypothetical protein	0.012
*Rv1816*	1.523189413	HTH-type transcriptional regulator	0.014
*Rv1990c*	1.503676645	Transcriptional regulator	1.23E-07

**FIG 2:**
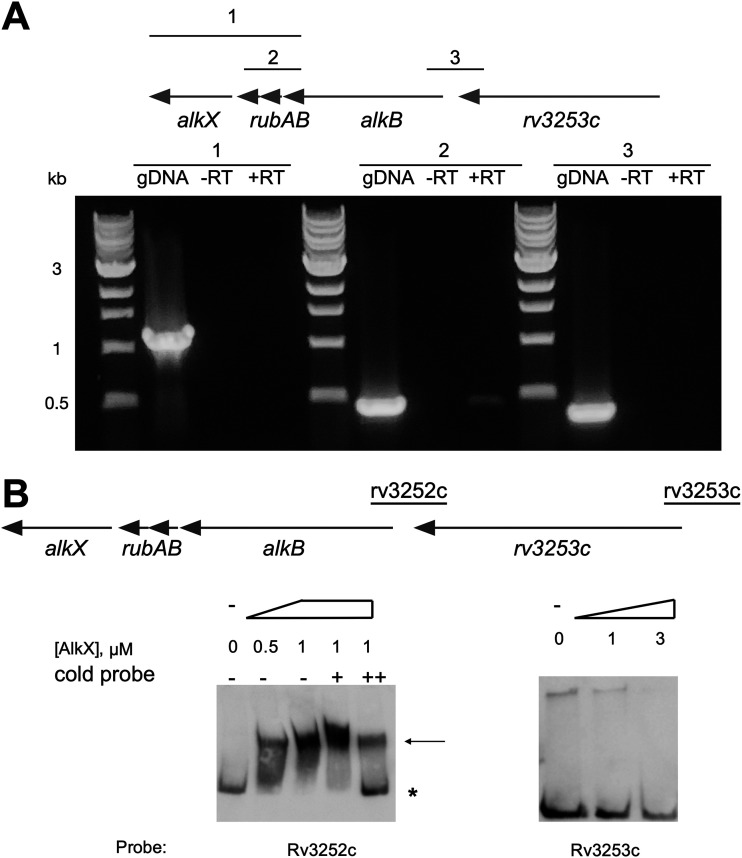
*alkB-rubAB* genes compose an operon that is directly regulated by AlkX. (A) PCR was used to amplify regions overlapping *rv3249c*-*3252c* (primer set 1), *rv3250c*-*rv3252c* (primer set 2), and *rv3253c-alkB* (primer set 3) in genomic DNA (gDNA), RNA (–RT, minus reverse transcription reaction), and cDNA (+RT) samples. cDNA template produced only a product with primer set 2, suggesting that only *alkB-rubAB* are cotranscribed. (B) EMSA analysis of AlkX demonstrated a concentration-dependent binding of AlkX with the promoter region upstream of *rv3252c* (*alkB*), but not that of *rv3253c*. Reactions were performed with 6 nM Dig-labeled probe and the indicated micromolar concentrations of protein. To demonstrate specificity, the Rv3252c EMSA was performed in the presence of nonlabeled (“cold”) probe at 120 and 240 nM (+ and ++, respectively). An arrow denotes the shifted probes, and the asterisk notes the accumulation of free Dig-labeled probe.

The *rv3250* to *rv3252c* genes encode the AlkB alkane-monooxygenase and rubredoxins A and B, and the adjacent *rv3253* gene encodes a putative cationic amino acid transporter. The *alkB-rubAB* genes have overlapping start and stop codons, suggesting that they are transcribed as an operon. There are only 108 bases between the stop codon of the *rv3253c* gene and the start codon of *alkB*. To determine if *alkB-rubAB* is cotranscribed, and assess whether *rv3253c* is also part of the operon, we performed reverse transcriptase PCR (RT-PCR). Our analysis confirmed that *alkB-rubAB* comprises an operon but that *rv3253c* is not cotranscribed (Fig. 2A, reactions 2 and 3). Despite the overlapping stop codon of *rubB* and *alkX*, we did not observe a product with RT-PCR for the *rubA-alkX* (reaction 1). This result may be due to small amounts of the transcript or an additional start site for *alkX* expression. Indeed, in addition to the transcription start site (TSS) upstream of *alkB*, we mapped a TSS for *alkX* within the *rubA* gene using 5′ rapid amplification of cDNA ends (RACE). This TSS is 205 nucleotides (nt) upstream of the *alkX* start codon and is included in our complementation vector.

To investigate binding of AlkX to the promoter of the AlkB-rubredoxin locus, we performed EMSA analysis. We observed a concentration-dependent shift of the *rv3252c* (*alkB*) promoter probe, but not the *rv3253c* promoter probe (Fig. 2B). As a control, we added increasing amounts of cold probe to the *alkB* EMSA. We observed release of the Dig-labeled probe consistent with specific binding of AlkX to the *alkB* probe. Combined, our results demonstrate that *alkB-rubAB* are cotranscribed and that AlkX directly controls the expression of the adjacent *alkB-rubAB* genes.

### The *alkX* mutant survives better than wild-type M. tuberculosis in macrophages.

**FIG 3:**
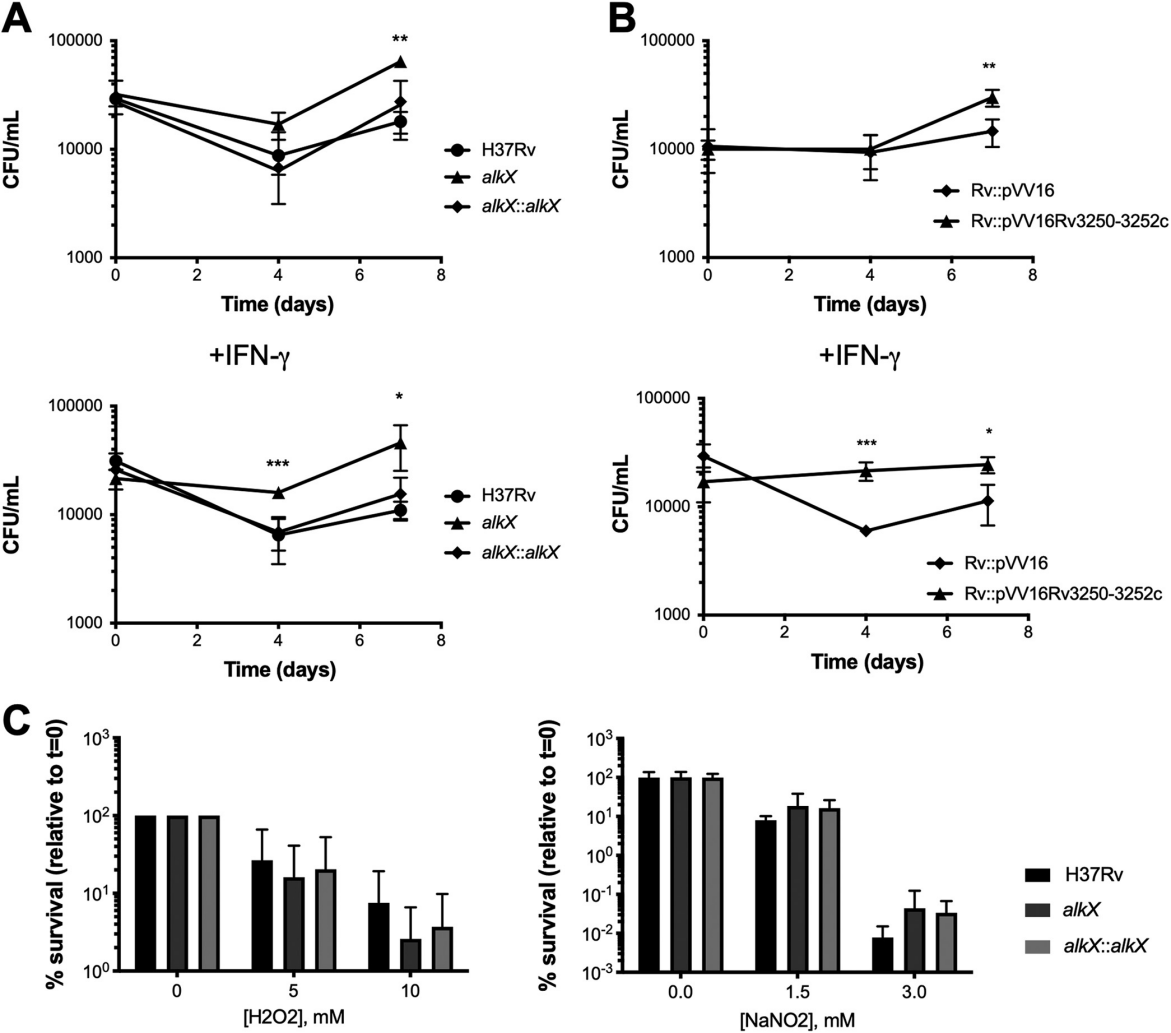
The *alkX* mutant grows better than wild-type M. tuberculosis in bone marrow-derived macrophages (BMMO). (A) BMMO were infected with wild-type, *alkX* mutant, and complemented M. tuberculosis strains. (B) BMMO were infected with wild-type and the AlkBRubAB OE strains. In panels A and B, viable bacteria were tracked over time by plating serial dilutions on 7H10 agar. The averages and standard deviations from a representative experiment performed in quadruplicate are shown. Student’s *t* test: *, *P* < 0.05; **, *P* < 0.01. (C) Wild-type, *alkX* mutant, and complemented M. tuberculosis strains were exposed to H_2_O_2_ and acidified NaNO_2_ at the indicated concentrations. Viable bacteria in treated and untreated samples were determined by plating serial dilutions on 7H10 agar. The averages and standard deviations from a representative experiment performed in triplicate are shown.

We investigated the fitness of the *alkX* mutant in the intracellular environment of the macrophage. We found that the *alkX* mutant survived better than wild-type M. tuberculosis in murine bone marrow-derived macrophages, and this phenotype was more pronounced in interferon-gamma (IFN-γ) activated macrophages (Fig. 3A). Complementation restored wild-type survival. This result suggests that overexpression of the AlkBRubAB system in the intracellular environment is advantageous to the bacterium. To directly test this possibility, we performed macrophage infections with wild-type M. tuberculosis (H37Rv/pVV16) and a strain that overexpresses *alkB-rubAB* from the strong, constitutive *hsp60* promoter of pVV16 (H37Rv/pVV16 *rv3250-rv3252c*). The overexpression strain survived and replicated better than wild-type M. tuberculosis in resting and activated macrophages (Fig. 3B). Combined, these results indicate that the AlkBRubAB rubredoxin system regulated by AlkX contributes to M. tuberculosis survival and replication in macrophages.

Macrophages, particularly activated macrophages, produce reactive oxygen intermediates (ROI) and reactive nitrogen intermediates (RNI) to target intracellular pathogens. Since the rubredoxins A and B were upregulated in the *alkX* mutant, we speculated that the *alkX* mutant may be better equipped than wild-type M. tuberculosis to counter ROI and RNI. To test this possibility, we performed *in vitro* assays exposing the bacterium to H_2_O_2_ as a source of ROI and acidified nitrite as a source of RNI. We did not observe significant differences between the *alkX* mutant and wild-type M. tuberculosis (Fig. 3C).

### The *alkX* mutant grows slower on fatty acids and glycerol.

**FIG 4:**
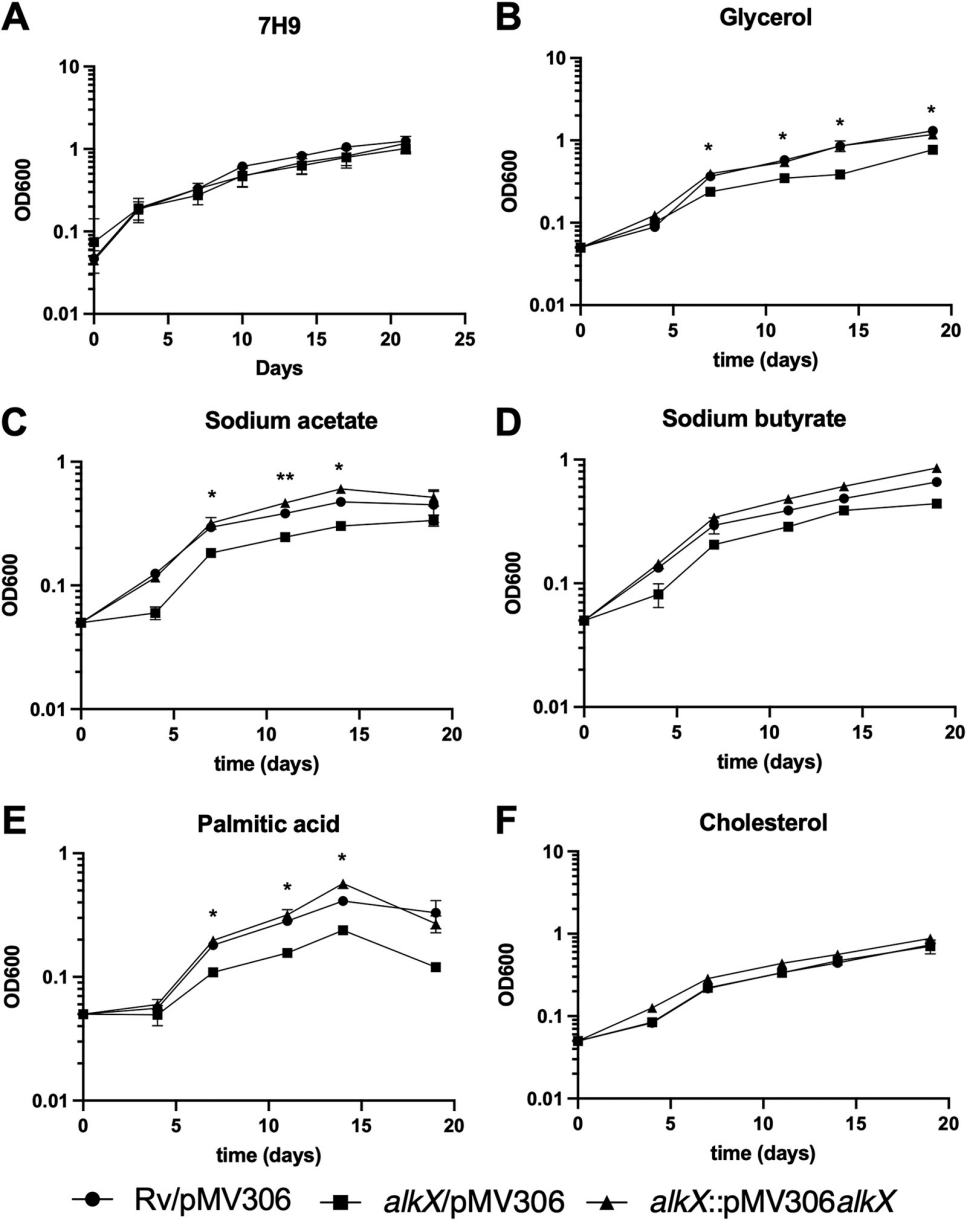
The *alkX* mutant had growth defects in specific carbon sources. (A) Replication of wild-type, *alkX* mutant, and complemented M. tuberculosis strains cultured in standard 7H9 ADS Tween medium. (B to F) Growth of the wild-type, *alkX* mutant, and complemented M. tuberculosis strains in minimal Sauton’s medium containing the indicated carbon source. Final concentrations used were 4.7% glycerol, 1 mM sodium acetate, 0.5 mM sodium butyrate, 0.05 mM palmitic acid, and 0.05 mM cholesterol. The average and standard deviation of an experiment representative of three biological replicates are shown. Paired Student’s *t* test: *, *P* < 0.05; **, *P* < 0.01.

Since the *alkB-rubAB* genes encode the alkane hydroxylase AlkB and rubredoxins A and B, an alternative explanation for increased survival in the macrophage could be increased growth in the host environment. However, we did not observe a growth phenotype for the *alkX* mutant compared to wild-type M. tuberculosis when the mutant was cultured in standard 7H9 ADS (albumin, desxtrose, saline) Tween medium (Fig. 4A). M. tuberculosis encounters and utilizes multiple host-associated carbon sources during infection, including fatty acids and cholesterol. To determine if loss of AlkX impacts bacterial growth on these carbon sources, we grew the wild-type, *alkX* mutant, and complemented strains in minimal media containing glycerol, acetate, butyrate, palmitate, or cholesterol. There was reduced replication on glycerol, acetate, and the long-chain fatty acid palmitate as the sole carbon sources (Fig. 4B, C, and E). On the other hand, the *alkX* mutant grew like wild-type M. tuberculosis on cholesterol as the sole carbon source (Fig. 4F). These combined results suggest that the *alkX* mutant has altered carbon metabolism but do not support the model where the increased fitness of the *alkX* mutant in macrophage infections stems from greater ability to utilize host-derived carbon sources.

### The *alkX* mutant has impaired biofilm formation associated with overexpression of AlkBRubAB.

**FIG 5:**
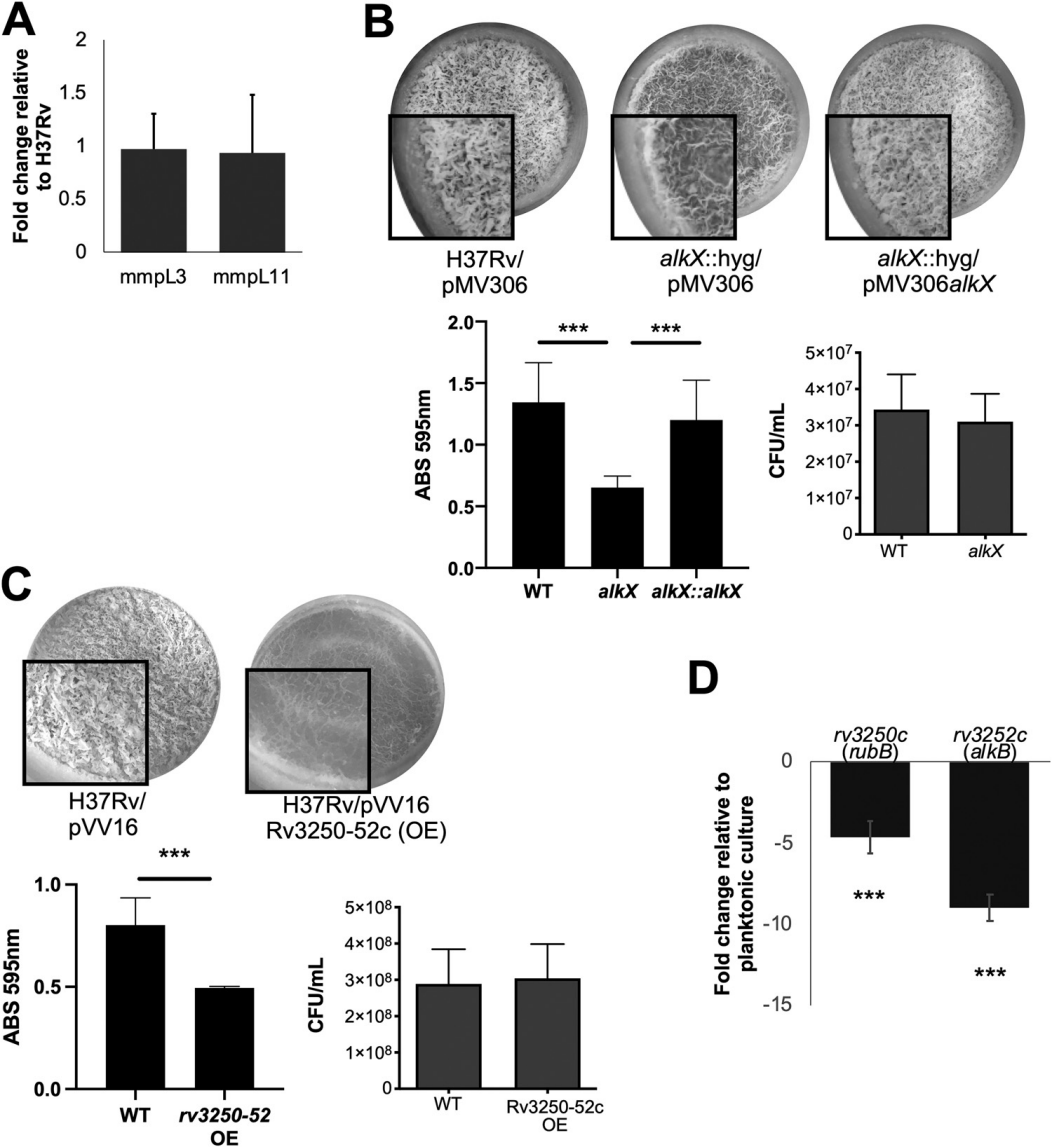
The *alkX* mutant has impaired biofilm formation. (A) qRT-PCR analysis showed no significant difference in the transcription of *mmpL3* and *mmpL11* between the mutant and wild-type strains when grown as biofilms. (B) Wild-type, *alkX* mutant, and complemented M. tuberculosis strains were cultured as biofilms and imaged. (C) The wild type and the strain overexpressing the *alkB-rubAB* operon were cultured in biofilms. In panels B and C, the biofilm material was quantified by CV staining and reported as absorbance at 595 nm. The number of bacteria present in the well were determined by mechanically disrupting the biofilm and plating serial dilutions on 7H10 agar. Student’s *t* test: ***, *P* < 0.005. (D) qRT-PCR analysis showed that *rubB* and *alkB* transcription is downregulated in biofilm-grown M. tuberculosis relative to planktonic bacteria. The difference between *rubB* and *alkB* expression in the biofilm cultures compared with planktonic cultures of M. tuberculosis was significant, Student’s *t* test: ***, *P* < 0.005. In all experiments, the averages and standard deviation of three biological replicates are shown.

We were initially interested in AlkX as a regulator of the lipid exporter MmpL11, which is required for biofilm formation (13, 16). While we did not observe an effect on *mmpL3* or *mmpL11* expression under planktonic conditions (Fig. 1C), it was possible that their expression was regulated differently under biofilm conditions. We therefore performed qRT-PCR to quantify *mmpL* gene expression in wild-type and *alkX* mutant strains. We did not observe significant differences between the strains (Fig. 5A). To determine if the *alkX* mutant had altered biofilm formation, we cultured wild-type H37Rv, the *alkX* mutant, and the complemented strain in Sauton’s medium lacking Tween. The *alkX* mutant had a visually thinner and weaker biofilm than both the wild type and the complemented strain (Fig. 5B). This corresponded with reduced CV staining of the *alkX* mutant biofilm material compared to wild-type and complemented strains. Quantification of viable bacteria in each biofilm culture confirmed that the defect was in pellicle formation rather than growth in Sauton’s medium.

We were curious if the overexpression of *alkB-rubAB* alone could recapitulate the biofilm phenotype of the *alkX* mutant. When H37Rv/pVV16 and H37Rv/pVV16*rv3250-52c* were grown as biofilms, the overexpression strain had a defect in biofilm formation similar to that of the *alkX* mutant (Fig. 5C). This corresponded with reduced CV staining of the overexpression strain biofilm material compared to wild-type strains. Since overexpression of *alkB-rubAB* was detrimental to biofilm formation, we investigated whether *rubB* and *alkB* expression was regulated in biofilm cultures relative to planktonic cultures. Using qRT-PCR, we found that *rubB* and *alkB* were downregulated in wild-type M. tuberculosis biofilms relative to planktonically grown bacteria (Fig. 5D). Taken together, our data suggest that overexpression of the AlkB-rubredoxin system impairs biofilm formation.

## DISCUSSION

This work defines the role of AlkX as a repressor of the *alkB-rubAB* locus. Upregulation of the AlkBRubAB system in the *alkX* mutant improved M. tuberculosis intracellular survival in resting and activated macrophages. Previous studies also support a role for the alkane hydroxylase system in the host. AlkB mutants were attenuated in a SCID mouse, indicating some role for this pathway during infection (17). Global transcriptomics showed that the *alkB-rubAB* genes were upregulated in resting and activated macrophages. *In vitro* stimulation with intracellular signals, such as H_2_O_2_ and the free fatty acid palmitic acid, also resulted in *alkB-rubA-rubB* upregulation (18). Expression of the *alkB* gene was also upregulated in an *in vitro* phosphate-buffered saline (PBS) starvation model, suggesting that the alkane hydroxylase system contributes to the bacterium’s adaptation to nutrient restriction (19).

AlkB is a predicted alkane hydroxylase. Alkanes are ubiquitous in nature and are produced by plants, algae, and other organisms in contaminated and noncontaminated environments. As such, the ability to oxidize and degrade alkanes is common in both Gram-positive and Gram-negative organisms (20). Environmental mycobacteria can degrade short alkanes; for instance, Mycobacterium vaccae JOB5 hydrolyzed propane and butane (21). While M. tuberculosis possesses AlkBRubAB, the ability of the pathogen to utilize alkanes has not been experimentally demonstrated. M. tuberculosis did not grow on the *n*-alkane paraffin, whereas M. avium-*intracellular* did, suggesting that this phenotype could distinguish nontuberculous mycobacteria (NTM) from M. tuberculosis in clinical specimens (7). Metabolomic analysis of M. tuberculosis strains identified alkanes and a glycolipid surfactant, d-glycero-l-mannoheptonic acid, that is implicated in facilitating alkane uptake (22). Therefore, the role of alkanes and alkane metabolism in M. tuberculosis remains unclear.

The gene encoding the AlkX transcriptional regulator is immediately downstream of the *alkB-rubAB* genes in M. tuberculosis. A similar genetic organization also exists for *alkB-rubAB* genes with a gene encoding a TetR family regulator in Gram-positive *Actinobacteria*, including *Rhodococcus*, *Nocardia*, and *Dietzia* (23). Recent work from Liang et al. demonstrated that the *Dietzia* DQ12-45-1bTetR regulator AlkX is a repressor of the locus analogous to M. tuberculosis *alkB-rubAB* (10). Interestingly, they showed that AlkX DNA-binding activity was reduced in the presence of palmitic acid. They highlighted this result as evidence for a product-positive feedback mechanism since long-chain fatty acids are generated as part of alkane degradation. Supporting this model, we previously showed that M. tuberculosis AlkX cocrystallized with palmitic acid, and addition of palmitic acid to EMSA reduced AlkX DNA-binding activity (13). Combined with our current results, our data support a generalized model where TetR repression of AlkBRubAB in *Actinobacteria* is subject to product-positive feedback via fatty acids.

## MATERIALS AND METHODS

### Bacterial strains and growth conditions.

The M. tuberculosis wild-type strain H37Rv was obtained from the ATCC. Mycobacterial strains are described in Table 2 and were routinely maintained in Middlebrook 7H9 liquid medium (Difco) with 0.05% Tween 80 or on Middlebrook 7H10 agar (Difco), both supplemented with albumin dextrose salts (ADS) containing 8.1 mg/mL NaCl, 50 mg/mL bovine serum albumin (BSA), and 20 mg/mL dextrose. Glycerol was added to liquid 7H9 to a final concentration of 0.5%. Kanamycin (25 μg/mL) and hygromycin (50 μg/mL) were used for selection when required. For growth on defined carbon sources, strains were grown without shaking in Sauton’s medium (0.5 g/L K_2_HPO_4_, 0.5g/L MgSO_4_, 4.0 g/L l-asparagine, 0.05 g/L ferric ammonium citrate, and 1.0 mg/L ZnSO_4_, with a final pH of 7.0, and 0.05% tyloxapol) containing glycerol, fatty acids, or cholesterol (Sigma). The final concentrations used were 4.7% glycerol, 1 mM sodium acetate, 0.5 mM sodium butyrate, 0.05 mM palmitic acid, and 0.05 mM cholesterol. Prior to addition, fatty acids were dissolved to 100 mM in a solution of tyloxapol/ethanol (1:1) at 80°C for 30 min and then added to the medium to a final concentration of 0.05 mM.

**TABLE 2:** Strains used

Strain	Genotype or description
H37Rv/pMV306kan	Wild-type H37Rv with the empty pMV306kan vector integrated
H37Rv/pVV16	Wild-type H37Rv transformed with the empty episomal vector pVV16
*alkX*/pMV306kan	*alkX* mutant with hygromycin resistance cassette inserted and with the empty pMV306kan vector integrated
*alkX*/pMV306kan*alkX*	*alkX* mutant with hygromycin resistance cassette inserted and with the pMV306kan vector containing *alkX* integrated
H37Rv/pVV16*rv3250-52*	Wild-type H37Rv transformed with the episomal vector pVV16 where the expression of *rv3250-52* is under the control of the strong *hsp60* promoter

The M. tuberculosis *rv3249c* (*alkX*) mutant was created via allelic exchange. Upstream and downstream regions of *alkX* were amplified by PCR using Δ*rv3249c* 5′/3′ forward/reverse primers (see Table 3 for all primer sequences) and cloned to flank the hygromycin resistance gene in pBSK. The resulting plasmid was linearized and used to transform electrocompetent H37Rv. Transformants were selected on 7H10 agar containing ADS and hygromycin (50 μg/mL). Deletion of *alkX* was confirmed via PCR using flanking primers followed by sequencing of the resulting PCR product. For complementation, *alkX *+ 568 bp upstream sequence was amplified via PCR with primers and cloned into the integrative vector pMV306kan. The sequence includes the TSS identified in the course of this work, as well as the coding sequence of *rubAB*. The resulting complementation plasmid was transformed into the M. tuberculosis *alkX* mutant and transformants selected on 7H10 agar containing ADS, hygromycin (50 μg/mL), and kanamycin (25 μg/mL).

**TABLE 3:** Primers used

Primer name	Sequence	Use^*a*^
5′ 3249c pMV306 compl KpnI	taggtaccactcgccgctatcagac	Complementing Rv3249c
3′ 3249c pMV306 compl stop HindIII	taaagctttcaggggacattgatcac	Complementing Rv3249c, RT-PCR analysis of Rv3252-3249c
Rv3249ckpn	atggtaccatcgatgatctcgctgacct	KO 5′
Rv3249cxho	atctcgaggagccacctccaccatctc	KO 5′
Rv3249cH3ko	ataagcttgcttgagctacgtgtcgatg	KO 3′
Rv3249cXba	attctagatggagaagatgttgcacgag	KO 3′
Rv3249c KO 5′ check	agaggccgtcaacta cctcga	KO check
Rv3249c KO 3′ check	acgccggccttctcatactgc	KO check
sigAF	tcgaggtgatcaacaagcctg	SigA qRT-PCR primer
sigAR	atggtctggtccaacgagat	SigA qRT-PCR primer
qAlkBF.221	cgcttcttgacctacgcttc	AlkB qPCR primer
qAlkBR.350	accacgctgaggtactggaa	AlkB qPCR primer
Rv3250cqF	caatgcggctttgagtacga	Rv3250c qPCR primer
Rv3250cqR	ccacctccaccatctcgaaa	Rv3250c qPCR primer
Rv3250-52_fwd	agtggtggtggtggtggtgaagcttcgagcgagccacctccaccatc	Cloning Rv3250-3252c locus for overexpression
Rv3250-52_rev	atccggaggaatcacttccatatgatgaccacgcaaatcggc	Cloning Rv3250-3252c locus for overexpression
Rv3253RTF	gtggctgatgctgaacctc	RT-PCR analysis of Rv3252-3253c
Rv3252RTR	cccataagccacaggtaacg	RT-PCR analysis of Rv3252-3253c
Rv3252RTF	actcgccgctatcagacact	RT-PCR analysis of Rv3252-3250c, Rv3252-3249c
Rv3250RTR	ctcaaagccgcattggatac	RT-PCR analysis of Rv3252-3250c
Rv3252F.emsa	tcggcaagctcgagagag	Rv3252c EMSA probe
Rv3252R.emsa	cttcgggtccaccagagc	Rv3252c EMSA probe
Rv3253F.emsa	tggagtagaagtccgagagca	Rv3253c EMSA probe
Rv3253R.emsa	cggcgattgactgttctacc	Rv3253c EMSA probe
alkBGSP2	catgccgaggctccgtaac	5′ RACE primer

a KO, knockout.

M. tuberculosis biofilms were grown in Sauton’s medium containing 0.5 g/L K_2_HPO_4_, 0.5g/L MgSO_4_, 4.0 g/L l-asparagine, 0.05 g/L ferric ammonium citrate, 2.0 g/L citric acid (anhydrous), 4.76% glycerol, and 1.0 mg/L ZnSO_4_, with a final pH of 7.0. Biofilms were inoculated to an optical density of 600 nm (OD_600_) of 0.05 in Sauton’s medium and incubated at 37°C/5% CO_2_ in tightly sealed polystyrene bottles or 50-mL conical tubes. At 2 weeks, the lids were loosened to permit gas exchange. Biofilms were imaged at 3 weeks postinoculation.

### RNA-seq.

Mycobacterial RNA was isolated as described previously (24). Briefly, bacteria were harvested by centrifugation, and the pellet was washed in GTC buffer (4 M guanidine thiocyanate, 0.5% Na *N*-lauryl sarcosine, 25 mM Na citrate, pH 7.0, 0.1 M β-mercaptoethanol) followed by phosphate-buffered saline (PBS)-0.1% Tween 80. Bacteria were disrupted by treatment with lysozyme followed by bead beating in the presence of warm TRIzol. RNA was subsequently isolated using DirectZol RNA isolation columns (Zymo). RNA was eluted in 50 μL RNase-free water and then treated with DNase (Ambion) to ensure removal of genomic DNA contamination.

RNA-seq analysis was performed at the Oregon State University Center for Genome Research and Biocomputing core facility. RNA libraries were prepared according to Illumina instructions. rRNA was removed using Ribo-Zero rRNA. Stranded RNA library prep was performed on a WaferGen Bio-systems Apollo 324 robot, and libraries were quantified by qPCR. RNA-seq was performed on an Illumina HiSeq 3000 instrument to produce 50-bp single-end reads. Illumina CASAVA v1.8 software used for base calling, and sequence reads were assessed for adapters and quality scores using FastQC. For each sample, sequence reads aligned to the M. tuberculosis H37Rv reference genome (GCF_000195955.2) and quantified using Salmon v1.4.0. Differential expression was assessed using DESeq2 v1.32.0. These data were submitted to the GEO repository, study GSE201641.

### qRT-PCR.

cDNA was prepared from 500 ng of RNA using the Bioline SensiFAST cDNA synthesis kit according to the manufacturer’s protocol. RNA and quality and concentration were measured on a NanoDrop ND-1000 spectrophotometer.

qRT-PCR was performed using a Bioline Sensifast Sybr HiRox kit with 100 ng of cDNA according to the manufacturer’s protocol. Samples were run in triplicate, and DNase-treated RNA was used as a negative control. Plates were sealed with ThermalSeal RT optically transparent sealing film (Excel Scientific). qRT-PCR was performed on a Bio-Rad CFX96 device, using the comparative threshold cycle (*C_T_*) method. The following protocol was used: initial denaturation at 95°C for 1 min and then a 2-step PCR with 40 cycles of 95°C for 5 s and 60°C for 30 s. Gene expression was normalized to that of *sigA*, and the fold change was calculated using the comparative *C_T_* method (25). Primers for qRT-PCR are listed in Table 3.

### Electrophoretic mobility shift assay.

Probes were amplified from the H37Rv genome using the primers listed in Table 3. All probes were labeled with digoxigenin using the Roche DIG gel shift kit. For EMSA analysis, 12 nM Dig-labeled probe and the indicated micromolar concentrations of protein were incubated for 45 min at room temperature in the Roche binding buffer modified by the addition of 0.25 mg/mL herring sperm DNA, and 0.75 mg/mL poly(d[I-C]). Reactions were resolved on a 6% native polyacrylamide gel in Tris-borate-EDTA (TBE) buffer and transferred to nylon membrane. Dig-labeled DNA-protein complexes were detected following the manufacturer’s recommendations. Chemiluminescent signals were acquired using an ImageQuant LAS 4000 system (GE).

### Determination of transcriptional start sites.

RNA was isolated from planktonic M. tuberculosis H37Rv as described above. Transcriptional start sites of *alkX* were elucidated using the Invitrogen 5′ RACE system for rapid amplification of cDNA ends v2.0. Briefly, 2 μg of DNAsed RNA was used to synthesize cDNA using a gene-specific antisense primer (pMV306 compl HindIII), which binds at the 3′ end of *alkX* (Table 3). Subsequently the RNA was degraded and cDNA was purified over a S.N.A.P column following the kit instructions. Terminal deoxynucleotide transferase (TdT) was used to add a 3′ C-tail to the cDNA, and tailed cDNA was amplified using an abridged anchor primer (provided by the kit) and an antisense gene-specific primer (alkBGSP2). The amplified sequence was cloned into a pGEM-T Easy Vector and transformed into DH5α. Sequencing of the cloned insert was performed using the T7 promoter primer.

### CV staining of biofilm material.

Biofilms were cultured in 50-mL conical tubes. At 3 weeks postinoculation, medium was removed using a pipette, and the tubes were washed twice with 5 mL PBS. After washing, 5 mL of 1% crystal violet (CV) was added to each tube and incubated for 15 min. CV was removed and washed twice with an equal volume of PBS. Following the washes, 95% ethanol was added to each tube, incubated for 10 min, and removed for analysis. Absorbance of the extracted CV was read at 595 nm with a plate spectrophotometer.

### Intracellular survival and bacterial replication in bone marrow-derived macrophages.

Bone marrow-derived macrophages (BMMO) were isolated from C57/Bl6 mice and maintained in Dulbecco’s modified Eagle’s medium (DMEM) (Gibco) supplemented with 10% fetal calf serum (FCS) (Gibco), 1.5 g/L sodium pyruvate (Gibco), and 20% L cell-conditioned medium. To activate BMMO, cells were treated with 10 ng/mL IFN-γ overnight. BMMO were infected at a multiplicity of infection (MOI) of 1:1 for 1 h and then washed with medium to remove extracellular bacteria. At the indicated time points, infected macrophages were lysed with 0.1% Tween 80, serially diluted, and plated on 7H10 agar plates to determine the CFU of the surviving M. tuberculosis. IACUC approval for bone marrow macrophage isolation is in place at OHSU.

### *In vitro* stress assays.

To determine the sensitivity to H_2_O_2_, early log cultures were normalized to 5 × 10^6^ CFU mL^−1^ and incubated for 4 h in the presence or absence of 5 mM and 10 mM H_2_O_2_. Each sample was serially diluted and plated onto 7H10 agar. For sensitivity to RNI, cultures were diluted 1:10 in 7H9 ADS Tween medium, pH 5.5 ± 1.5 mM and 3 mM NaNO_2_ and incubated for 6 days following an established protocol (26). Each sample was serially diluted and plated onto 7H10 agar to determine the number of surviving bacteria.
